# Changes of Ammonia-Metabolizing Enzyme Activity and Gene Expression of Two Strains in Shrimp *Litopenaeus vannamei* Under Ammonia Stress

**DOI:** 10.3389/fphys.2018.00211

**Published:** 2018-03-23

**Authors:** Liguo Qiu, Xiang Shi, Simeng Yu, Qian Han, Xiaoping Diao, Hailong Zhou

**Affiliations:** ^1^State Key Laboratory of Marine Resource Utilization in South China Sea, Hainan University, Haikou, China; ^2^Institute of Tropical Agriculture and Forestry, Hainan University, Haikou, China; ^3^Lingcheng 6th Middle School, Dezhou, China

**Keywords:** *Litopenaeus vannamei*, ammonia-N stress, IBR, enzyme activities, gene expression

## Abstract

Ammonia stress can inhibit the survival and growth, and even cause mortality of shrimp. In this study, ammonia-metabolizing enzyme activities and gene expression were compared between two strains of *L. vannamei* under different ammonia-N (${\text{NH}}_{4}^{+}$) concentrations (3.4, 13.8, and 24.6 mg/L). The results showed that elevated ammonia concentrations mainly increased glutamine synthetase (GSase) activities while inhibiting transglutaminase (TGase) activities in the muscle of both strains. Thus, we concluded that *L. vannamei* could accelerate the synthesis of glutamine from glutamate and ${\text{NH}}_{4}^{+}$ to alleviate ammonia stress. Compared with the muscle, the hepatopancreas plays a major role in ammonia stress and might be a target tissue to respond to the ammonia stress. Compared to the control group, the treatment of high ammonia concentrations reduced the hepatopancreas TGase (*TG*) gene expression and increased the gene expression rates of glutamate dehydrogenase-β (*GDH*-β) and GSase (*GS*) in both the muscle and the hepatopancreas of the two strains (*p* < 0.05). These genes (*GDH*-β and *GS*) in strain B were not only expressed earlier but also at levels higher than the expression range of strain A. At the gene level, strain B showed a more rapid and positive response than strain A. These data might help reveal the physiological responses mechanisms of shrimp adapt to ammonia stress and speed up the selective breeding process in *L. vannamei*.

## Introduction

Due to its high commercial value, the white-legged shrimp (*Litopenaeus vannamei*, Boone, 1931) has been widely cultured throughout the world (Wu et al., 2008). With the rapid increase of intense cultivation, aquaculture has been impacted by complex mixtures of various contaminants, especially ammonia-N, which can drastically degrade the function of marine ecosystems (Zhang et al., 2009). As a primary environmental factor, ammonia-N can rapidly increase mortality and lead to severe economic losses in shrimp cultivation industry (Cobo et al., 2014). Previous studies have also shown that various tissues of *L. vannamei* had been seriously affected when exposed to different ammonia stress levels (Racotta and Hernández-Herrera, 2000; Liang et al., 2016; Liu et al., 2016; Zhou et al., 2017).

In China, many shrimp farms must import rapidly growing *L. vannamei* from several foreign companies. Compared with native shrimp, the progeny of the imported parent prawns adapts poorly to local conditions, resulting in suboptimal farming conditions (Briggs et al., 2004). In recent years, studies have mainly focused on the effects of temperature stress (Zhou et al., 2011), osmotic stress (Liu et al., 2012), viral infections (Song et al., 2003), salinity (Silvia et al., 2004), and acute hypoxia (Wei et al., 2016). However, few studies have investigated ammonia-metabolizing enzymes in different tissues of local strains of *L. vannamei* in response to ammonia stress. To explore the metabolic responses mechanism of *L. vannamei* under the ammonia stress, two shrimp strains with different susceptibility to ammonia were evaluated in this study. Additionally, the activity of three ammonia-metabolizing enzymes, including glutamate dehydrogenase (GDHase), glutamine synthetase (GSase), transglutaminase (TGase), and their gene expression (*GDH*-β*, GS*, and *TG*) were investigated in muscle and hepatopancreas tissues under exposure to different ammonia levels.

**Figure 1:**
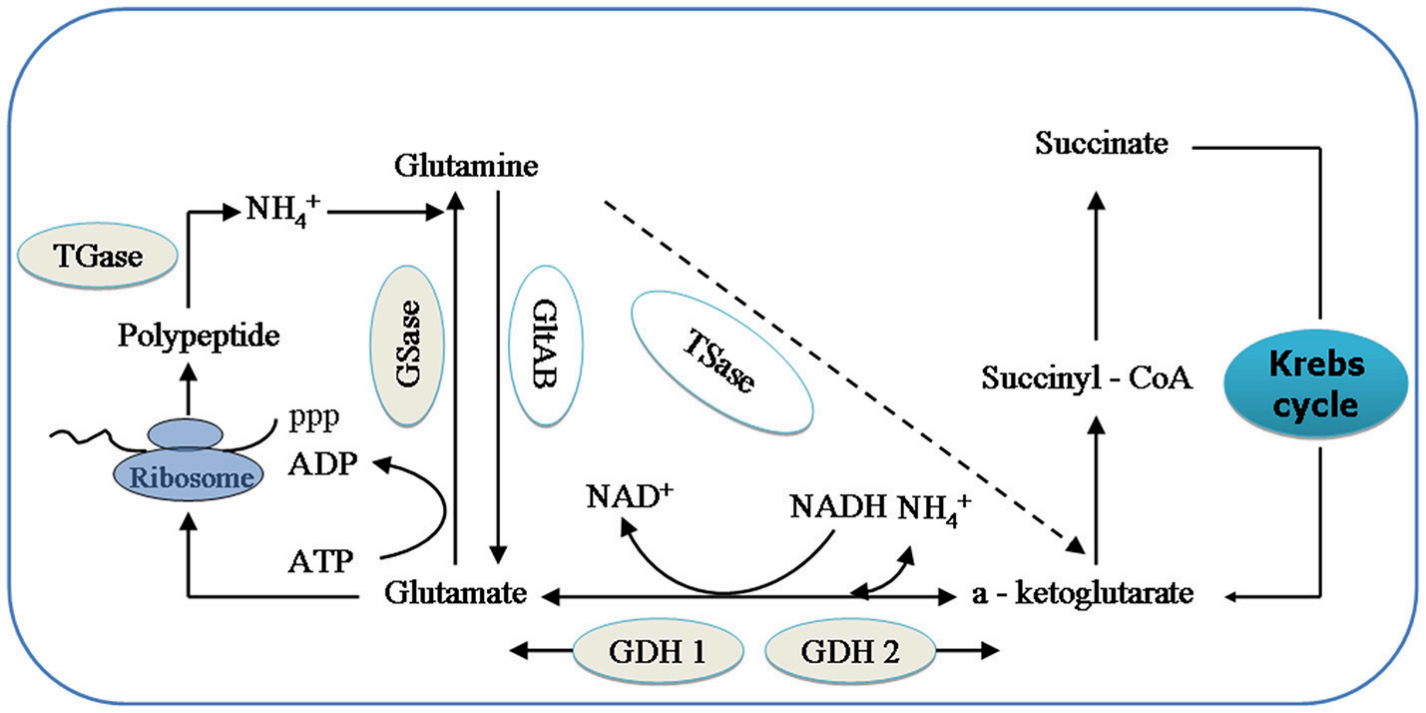
The relationship between relative enzymes and ammonia (${\text{NH}}_{4}^{+}$). Key intermediates and enzymes related to synthesize and catalyze are shown. GSase, glutamate synthase; TSase, Transaminase; GDH 1, NADH—dependant—glutamate dehydrogenase; and GDH 2, NAD^+^- dependant glutamate dehydrogenase; GSase, glutamine synthetase and TGase, Transglutaminase.

GDHase is the key enzyme in the oxidative reaction of amino acids through transdeamination in shrimp (Mayzaud and Conover, 1988). GDHase contains two parts, NADH-dependent glutamate dehydrogenase (GDH 1) and a NAD^+^-dependent subunit (GDH 2). The GDH 1 can catalyze a-ketoglutarate and ${\text{NH}}_{4}^{+}$ to synthesize glutamate while the GDH 2 catalyzes the reversible reaction (Figure 1; Cooper, 2012). GSase plays an essential role in the metabolism of nitrogen by catalyzing the ${\text{NH}}_{4}^{+}$ and glutamate to synthesize glutamine (Kosenko et al., 2003; Essex-Fraser et al., 2005). Former studies have shown that TGase is an essential component in the shrimp immune system (Yeh et al., 2009; Fagutao et al., 2012). The major catalytic routes of the enzymes with ammonia (${\text{NH}}_{4}^{+}$) are shown in Figure 1.

The reaction of biochemical endpoints in the form of biomarkers can provide valuable information on the mechanisms of toxic compounds (Hagger et al., 2009). However, finding a proper method to analyse the integrated biomarkers is a key challenge (Kim et al., 2010). Given that the activity of a single enzyme is irregular with the increase in ammonia, the IBR method was used to analyse all enzyme activities. Then, the IBR index, which uses star plots to summarize biomarker responses into a single value, was utilized to clearly reflect the levels of the induced enzyme activities under ammonia stress (Beliaeff and Burgeot, 2002). In this study, the corresponding gene (*GDH*-β*, GS*, and *TG*) expressions were also detected using Real-time reverse transcription PCR (qRT-PCR). The purpose of this work is to reveal the ammonia metabolic regularity mechanism using the pivotal enzyme (GDHase, GSase, and TGase) and the expression characteristics of crucial genes in different strains of *L. vannamei* under ammonia stress.

## Materials and methods

### Animals and experimental design

Two strains of *L. vannamei* (7.5 ± 0.5 cm, 3.8 ± 0.6 g) were obtained from the Guangtai shrimp farm in Hainan Province, China and acclimatized for 10 days in seawater tanks (Salinity 31%0, T 26 ± 1°C, pH 8.1 ± 0.5). Our previous studies have shown that the ammonia-N tolerance of strain B 3,271 (B) is better than that of strain A 3,281 (A). The shrimp (80 L of seawater per 30 individuals) were exposed to 0, 10, and 20 mg/L ammonia-N for 0, 5, and 10 days (Lin and Chen, 2001; Liu and Chen, 2004; Li et al., 2007). The actual mean concentrations in the control and experimental groups were 3.4, 13.8, and 24.6 mg/L, as measured by an ammonia meter (HI96733, HANNA). Each concentration was conducted in triplicate, and the water quality was measured twice each day. The ammonia-N solution was prepared using dissolved NH_4_Cl (A.R), as reported by Liu and Chen (2004). During the exposure periods, shrimp were fed with a formulated shrimp diet twice a day, and half of the seawater in the tank was replaced once a day. After collection, all muscles and the hepatopancreas of each shrimp in all experimental treatments were dissected, frozen in liquid nitrogen, and stored at −80°C until further analysis. No mortality was observed during the experiment.

### The GDHase, GSase, and TGase activity assays

The GDHase assay was performed on crude homogenates of each tissue following the method by Regnault (1993). The GSase activity was measured on the basis of γ-glutamyl transfer reaction (Woolfolk et al., 1966). The TGase activity was measured using the method previously described by Liu et al. (2011). Briefly, the tissues were homogenated at a ratio of 1:10 (w:v) at a normal salinity at 4. Then, the samples were centrifuged at 3,000 rcf for 10 min at 4°C. The optical densities of the samples were measured at 340 nm for GDHase, 540 nm for GSase and 450 nm for TGase. One unit of each active enzyme was defined as one gram of tissue in each reaction system (ml), to make the variation of the light absorption value 0.01 under the specific light wave length per minute.

### Integrated biomarker response

The IBR was calculated according to Beliaeff and Burgeot (2002). Briefly, the biomarker data of the enzyme activities were standardized, and the scores were represented in star plots. Then, the data from the biomarkers (GDHase, GSase, and TGase) were introduced to the IBR calculation. Star plots were used to visualize the respective scores for each enzyme (**Figures 4**, **5**). The total area corresponding to a given enzyme activity (IBR value) was obtained as:

$$\begin{array}{l} IBR=\overset{n}{\underset{i=1}{\sum }}Ai \end{array}$$

where *A*_*i*_ = S _*i*_ / 2 sin β (S_*i*_ cos β + S _*i*__+__1_ sin β), β = *Arc* tan (S_i+1_ sin α/ S_*i*_ – S_i+1_ cos α), α = 2 π / 3, S_*i*__+__1_ = S _1_.

### Total RNA extraction, reverse transcription, and qRT-PCR analysis in tissues of strains A and B

The extraction of total RNA and reverse transcription, as well as the qRT-PCR, were performed per the methods of our previously paper (Wei et al., 2016). *GDH*-β,*GS* and *TG* were determined for each cDNA using qRT-PCR on a ROCHE Light Cycler 96 Real-Time Cycler PCR Detection System (Roche Applied Science, Mannheim, Germany) using the following specific primers, Forward/Reverse sequence for *GDH*-β (5'−3') (CTTTCCAGGATCGCATTTCT; AAGCAG CAGTACGGAGATCAA) and *GS* (GGCATGGAGCAGGAGTA; CGCCGCAGT AGTAGGGT); for *TG* (CCTCAGGATCTCCTTCACCA; TTGGGAAAACCTTCA TTTCG); as well as the β-actin (CGCGACCTCACAGACTACCT, GTGGTCATC TCCTGCTCGAA) (Li and Wormhoudt, 2015; Liu et al., 2016). The thermal cycling conditions for the qRT-PCR consisted of denaturation at 95°C for 15 min, followed by 40 cycles at 95°C for 15 s, 60°C for 30 s then a final hold at 60°C for 60 s (Wang et al., 2010). For each cDNA sample, ΔCt was calculated from the threshold PCR cycle (Ct) of the test gene normalized relative to the Ct of β-actin in the same sample.

### Statistical analysis

A one-way analysis of variance (ANOVA) and multiple comparisons were performed using SPSS 19.0 with *p* < 0.05 showing significance. All figures were drawn by Origin 8.0 and Graphpad prism 5.0.

## Results

### Effects of ammonia-N on GDHase, GSase, and TGase activities in the muscles of strains A and B

**Figure 2:**
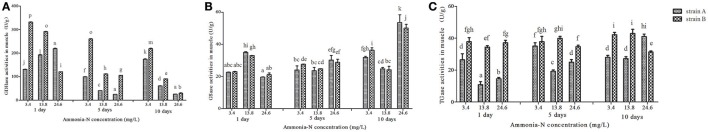
The activities (Mean ± SE) of GDHase, GSase and TGase in the muscle of strains A and B of *L. vannamei* in 1, 5 and 10 days exposure to different concentrations of ammonia-N. Each bar represents mean value from three determinations with standard error. Data in the same exposure time with different letters are significantly different (*p* < 0.05) between treatments. **(A)** GDHase activity in the muscle of strain A and B. **(B)** GSase activity in the muscle of strain A and B. **(C)** TGase activity in the muscle of strain A and B. Keys: GDHase, Glutamate dehydrogenase; TGase, Transglutaminase; GSase, Glutamine synthetase.

Ammonia stress (3.4–24.6 mg/L) had a significant effect on the selected enzyme activities in the muscles from both strains, and the results are shown in Figure 2. The activity of GDHase in the muscles from the two families showed a tendency to decrease with the increase in ammonia-N concentration (Figure 2A). Compared with strain A, the GDHase activity in the muscle from strain B was higher (Figure 2A). However, compared with the control, no significantly different GSase activity was found within 5 days in the muscle of the two strains (*p* > 0.05; Figure 2B). On the last day, the GSase activities increased with the increase in ammonia stress in the muscles of both strains (*p* < 0.05; Figure 2B). Compared to the control group, the TGase activity was significantly lower after 5 days in both strains. On the tenth day, the TGase activity continued decreasing in strain B, while increasing in the muscle of strain A (Figure 2C).

### Effects of ammonia-N on GDHase, GSase, and TGase activities in the hepatopancreas of strains A and B

**Figure 3:**
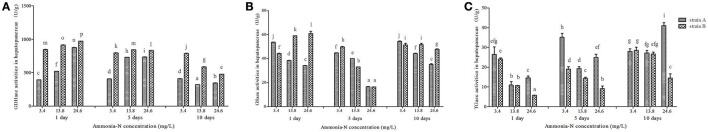
The activities (Mean ± SE) of GDHase, GSase and TGase in the hepatopancreas of strains A and B of *L. vannamei* in 1, 5 and 10 days exposure to different concentrations of ammonia-N. Each bar represents mean value from three determinations with standard error. Data in the same exposure time with different letters are significantly different (*p* < 0.05) between treatments. **(A)** GDHase activity in the hepatopancreas of strain A and B. **(B)** GSase activity in the hepatopancreas of strain A and B. **(C)** TGase activity in the hepatopancreas of strain A and B. Keys: GDHase, Glutamate dehydrogenase; TGase, Transglutaminase; GSase, Glutamine synthetase.

Ammonia exposure significantly affected the hepatic GDHase activities in two strains of *L. Vannamei* (*p* < 0.05; Figure 3). With the increase in ammonia concentration, the hepatic GDHase activity index increased by 50% after 5 days in both strains (Figure 3A). On the last day, the GDHase activity decreased in both strains with the increase in ammonia concentration (Figure 3A; *p* < 0.05). Similar to the variation in the muscles, the GDHase activity in strain B was higher than that of strain A in almost every ammonia exposure group (*p* < 0.05). Contrary to the muscle tissue, the hepatic GSase activity decreased compared to the control group with the increase in ammonia stress in almost all groups of both strains (*p* < 0.05; Figure 3B). Compared to the control group, a significant decrease in TGase activity was detected in the hepatopancreas from strain B with the increase of ammonia-N (*p* < 0.05) (Figure 3C). However, in strain A, the TGase activity first decreased then increased after 5 days when the concentration of ${\text{NH}}_{4}^{+}$ increased in the hepatopancreas (Figure 3C). Finally, the hepatic TGase activities increased with the increase of ammonia stress in strain A (Figure 3C).

### The IBR index analysis of three enzymes in the muscles and hepatopancreas of strains A and B

**Figure 4:**
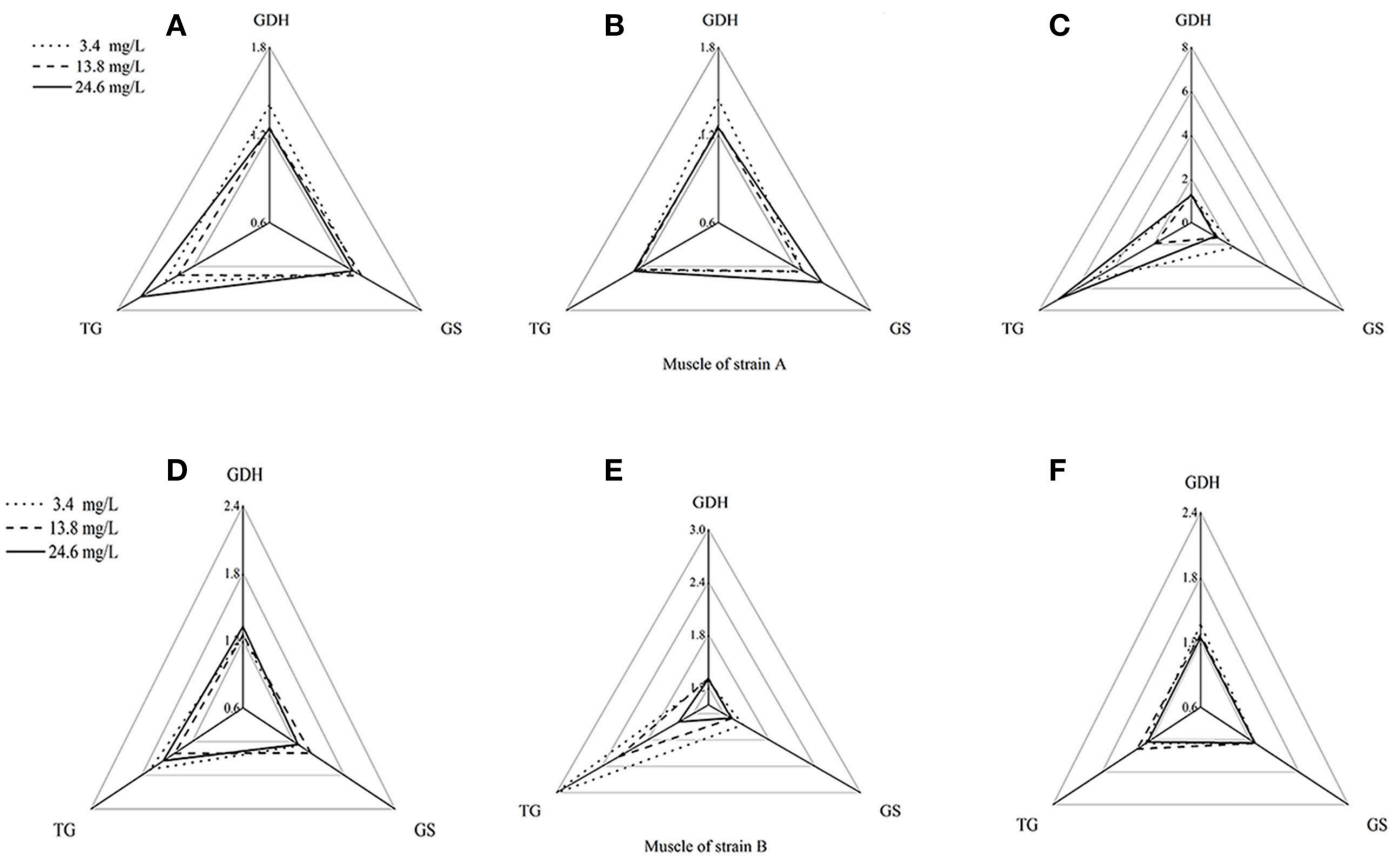
Star plots for biomarkers response in muscles of strains A and B exposed to different ammonia-N concentrations. The relative area of triangles (clockwise direction) indicates the affected degree of ammonia-N to GDHase, TGase and GSase, respectively. **(A)** Star plots for biomarkers response in muscle of strain A under ammonia-N pollution stress for the first day. **(B)** Star plots for biomarkers response in muscle of strain A under ammonia-N pollution stress for the fifth day. **(C)** Star plots for biomarkers response in muscle of strain A under ammonia-N pollution stress for the tenth day. **(D)** Star plots for biomarkers response in muscle of strain B under ammonia-N pollution stress for the first day. **(E)** Star plots for biomarkers response in muscle of strain B under ammonia-N pollution stress for the fifth day. **(F)** Star plots for biomarkers response in muscle of strain B under ammonia-N pollution stress for the tenth days. Keys: GDHase, Glutamate dehydrogenase; TGase, Transglutaminase; GSase, Glutamine synthetase.

**Figure 5:**
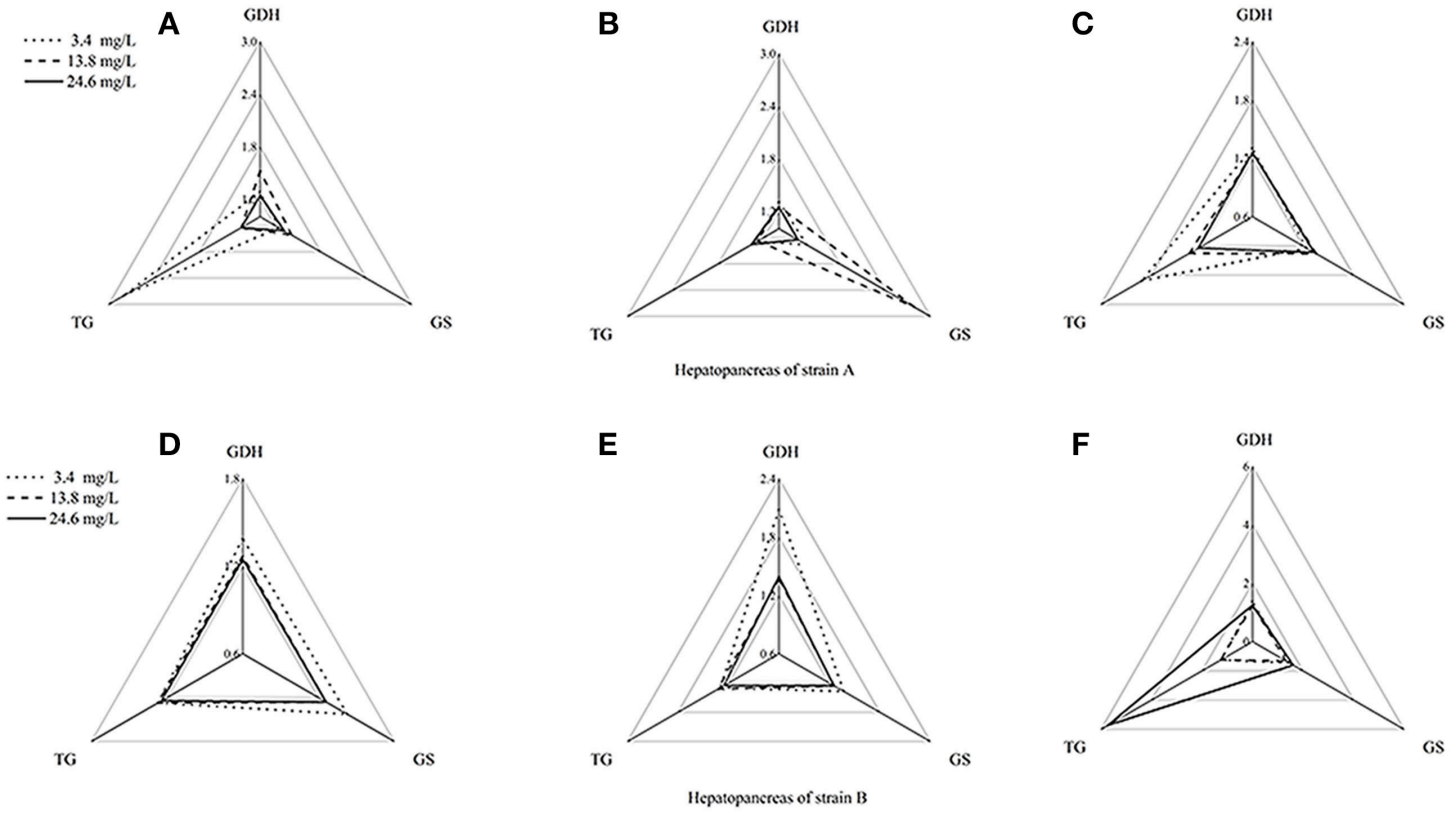
Star plots for biomarker responses in hepatopancreas of strain A exposed to different ammonia-N concentrations. The relative area of triangles (clockwise direction) indicated that the affected degree of ammonia-N to GDHase, TGase and GSase, respectively. **(A)** Star plots for biomarkers response in hepatopancreas of strain A under ammonia-N pollution stress for the first day. **(B)** Star plots for biomarkers response in hepatopancreas of strain A under ammonia-N pollution stress for the fifth day. **(C)** Star plots for biomarkers response in hepatopancreas of strain A under ammonia-N pollution stress for the tenth day. **(D)** Star plots for biomarkers response in muscle of strain A under ammonia-N pollution stress for the first day. **(E)** Star plots for biomarkers response in muscle of strain A under ammonia-N pollution stress for the fifth day. **(F)** Star plots for biomarkers response in muscle of strain A under ammonia-N pollution stress for the tenth day.

**Figure 6:**
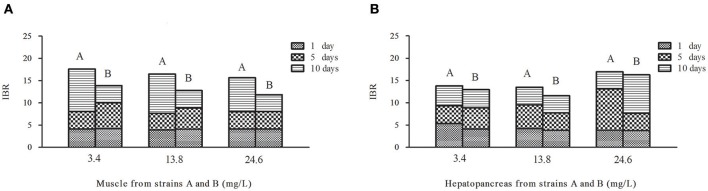
Temporal variation of IBR values in muscles **(A)** and hepatopancreas **(B)** of strains A and B under the stress of ammonia-N pollution. The word (A or B) above the column means strain A or strain B.

Using the standardization procedure described previously, star plots were used to place coordinates on star plot radii to represent biomarker data graphically (Figures 4, 5). Corresponding to the areas, the three directions on the star map represent the biomarkers (GDHase, GSase, and TGase) and the area of the polygon indicates the IBR value. Figure 6 shows the quantitative relationships between the total IBR values of the three biomarkers for the two tissues from both strains. The figure displays the trends of the chosen biomarkers when the ambient concentration of ammonia changed.

Figure 4 showed a reasonable visual agreement between the ammonia concentrations and the biomarkers after 1, 5, and 10 days in the muscles from the two strains. The areas of the triangles indicated the degree of influence that the biomarkers suffered after exposure to different concentrations of ${\text{NH}}_{4}^{+}$. From the corresponding star plots, no significantly different biomarkers were found after 5 days for strain A (Figures 4A,B). When the ammonia increased, the IBR values of GSase and TGase tended to increase, which can be observed directly from the increased area of the triangle on the tenth day (Figure 4C). In the muscle, these data suggested that the GSase and TGase activities in strain A are more influenced by the increase in ammonia stress. In strain B, the IBR index clearly displayed stress levels of GSase and TGase biomarkers, showing a high value in the muscle of strain B after 5 days (Figures 4D,E). However, the IBR values of the biomarkers appeared to decrease from 5 to 10 days in the muscle of strain B (Figure 4F). In the muscles, the same biomarkers (GSase and TGase) were found to be induced by ammonia stress in both strains. The reactive time in strain B (1 < T < 5 days) (T is exposure duration time) was much earlier than in strain A (5 < T < 10 days; Figure 4).

The biomarker responses in the hepatopancreas of strain A were completely different in response to ammonia exposure (Figures 5A–C). Compared with the IBR values, there was no substantial induction of biomarkers under different concentrations of ammonia-N, which can be clearly seen from the different areas of the triangles (Figures 5A–C). Special induced biomarkers were found in the hepatopancreas of the strain after increased exposure time (Figures 5D–F). Compared to the first day (Figure 5D), no significant differences in the biomarkers were detected in the hepatopancreas of strain B with the increase of ammonia-N stress after 5 days (Figure 5). However, similar to the results in strain A (Figure 4), two induced biomarkers (GSase and TGase) were detected in the 24.6 mg/L group (Figure 5F).

### The total IBR index analysis in the muscles and hepatopancreas between strains A and B

Contrary to the hepatopancreas (Figure 6B), the IBR response was down-regulated in the muscle (Figure 6A) with the increase of ammonia-N. Given that the total IBR value of the biomarkers can be an indicator of environmental stress, hepatopancreas tissues appear to bear more stress with the increase of ammonia-N stress. Meanwhile, the IBR values showed that the muscle tissue in strain A was significantly affected on the tenth day, while the impact occurred much earlier in strain B (in 5 days) (Figure 6A). When comparing the two strains, we found that the total IBR values of strain A were higher than that of strain B in both tissues of every ammonia concentration group. These data indicated that strain A was more sensitive to ammonia stress and the data agreed with the actual strain susceptibility, which also confirmed that the selected enzymes might be suitable biomarkers for ammonia exposure.

### Effects of ammonia-N on *GDH-β, GS*, and *TG* gene expression in muscles of strains A and B

**Figure 7:**
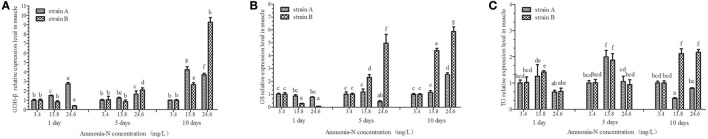
The effect of ammonia-N on the expression of *GDH*-β,*GS*, and *TG* genes expression in muscles from strains A and B. Different letters indicated significant differences at the level of 0.05. **(A)** *GDH*-β gene relative expression level in the muscle of strain A and B. **(B)** *GS* gene relative expression level in the muscle of strain A and B. **(C)** *TG* gene relative expression level in the muscle of strain A and B. Keys: *GDH*, Glutamate dehydrogenase; *TG*, Transglutaminase; *GS*, Glutamine synthetase.

The expressions of the *GDH*-β and*GS* genes were significantly affected by the increase of ammonia-N in the muscle of strains A and B (*p* < 0.05) and the expression increased (Figures 7A,B). Compared to the control group, significantly increased *GDH*-β gene expression (*p* < 0.05) was detected in the muscles of both strains from 5 to 10 days with the increase of ammonia-N (Figure 7A). Compared to strain A, the highest rate of expressed *GDH*-β (approximately 10-fold) was found in the muscle of strain B in the 24.6 mg/L ammonia-N group (Figure 7A).

Compared to the control group, the GS expression was significantly decreased (*p* < 0.01) after 5 days with the increase of ammonia-N in strain A (Figure 7B). A significant increase in *GS* expression was detected in the muscle from strain A after 5 days of exposure (Figure 7B). Compared to the expressed time, the up-regulation of *GS* expression was much earlier in strain B (1 < T < 5 days) than in strain A (5 < T < 10 days) under ammonia-N stress in the muscle. The expressed level of *GS* in the muscles of strain B are much higher than in strain A (*p* < 0.01), which can be seen clearly on the fifth and tenth days (Figure 7B).

No significant differences in *TG* expression (*p* > 0.05) was detected in the muscles between strains A and B under the same ammonia-N exposure concentration after 5 days (Figure 7C). Compared to the control group, the expression of *TG* appeared to be opposite in the two strains (Figure 7C). In the muscles, the *TG* expression increased approximately 2-fold in strain B, while it decreased approximately 0.5-fold in strain A by the tenth day (Figure 7C).

### Effects of ammonia-N on *GDH-β, GS*, and *TG* gene expressions in the hepatopancreas of strains A and B

**Figure 8:**
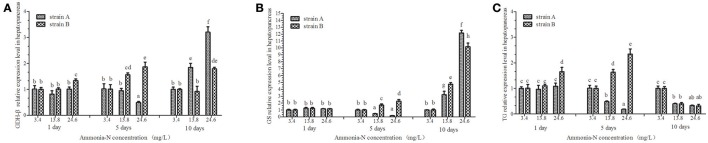
The effect of ammonia-N on the expression of *GDH*-β,*GS*, and *TG* genes expression in hepatopancreas from strains A and B. Different letters indicated significant differences at the level of 0.05. **(A)** *GDH*-β gene relative expression level in the hepatopancreas of strain A and B. **(B)** *GS* gene relative expression level in the hepatopancreas of strain A and B. **(C)** *TG* gene relative expression level in the hepatopancreas of strain A and B. Keys: *GDH*, Glutamate dehydrogenase; *TG*, Transglutaminase; *GS*, Glutamine synthetase.

Ammonia stress had a significant effect on the *GDH*-β expression in the hepatopancreas of strain B and induced its expression (*p* < 0.01; Figure 8). Compared to the control, although *GDH*-β expression was induced under the ammonia-N stress in the hepatopancreas of strain A, the changes were not significant on the first day (Figure 8A). The hepatic *GDH*-β gene decreased with increased ammonia-N stress on the fifth day and then induced on the last day in strain A (Figure 8A). Similar to the *GDH*-β gene expression in the muscles (Figure 7A), compared to the control group, the expression of *GDH*-β increased significantly with the increase of ammonia-N stress on the last day in the hepatopancreas of both strains (*p* < 0.01; Figure 8A).

Ammonia stress had a significant effect on the *GS* expression in the hepatopancreas of strains A and B, and the expression was up-regulated (Figure 8B). Significant differences in *GS* expression were detected in the hepatopancreas between the two strains after 5 days (Figure 8B; *p* < 0.01). Specifically, similar to the expression of *GDH*-β (Figure 8A) on the fifth day, the *GS* gene expression decreased in strain A, while it increased significantly in strain B under ammonia-N stress (Figure 8B). This indicated that the induced *GDH*-β and *GS* genes might help decompose the ammonia-N in strain B. Compared with the control, increased GSase expression was detected in the hepatopancreas from both strains A (approximately 12-fold) and B (approximately 10-fold) with the increase of ammonia-N (Figure 8B; *p* < 0.01).

The hepatic *TG* gene was found to be supressed in both strains, and its expression decreased under ammonia stress (Figure 8C). In the hepatopancreas, the expression of *TG* was found to decrease on the fifth day in strain A with the increase of ammonia-N (Figure 8C). The down-regulated *TG* expression was also detected on the tenth day in strain A, and no significant difference was found between the 13.8 and 24.6 mg/L groups (Figure 8C). Interestingly, the induced hepatic *TG* expression was detected in strain B after 5 days with the increase of ammonia-N (Figure 8C). On the tenth day, the same down-regulated *TG* gene expression was observed in both strains A and B under ammonia stress, and no significant difference existed between the two strains (Figure 8C). More studies are needed to explain the physiological responses mechanism of the *TG* gene expression among different strains of *L. vannamei*.

## Discussion

When exposed to an enriched ammonia environment, tissues in cultured shrimps have unique metabolic pathways for ammonia metabolism (Chang et al., 2015). Enzymatic activities can be applied as specific and fast biomarkers to identify toxic effects in shrimp (Wang et al., 2012). In most cases, studies only focused on the activities of irrelevant enzymes involved in the immune response, but not the ammonia-metabolizing enzyme (Li et al., 2016). In this study, three ammonia-metabolizing enzymes, including a synthetase enzyme (GSase), a degrading enzyme (TGase) and one neutral enzyme (GDHase), were chosen as biomarkers to detect the toxicological effects of ammonia. Similar to our previous study, we tested the susceptibility of the two strains in the experiment and found strain A was more sensitive to ammonia stress than strain B (Wei et al., 2016).

### The temporal and spatial variation of enzyme activities and gene expression response to ammonia stress

In most cases, both the GDHase activity and *GDH* gene expressions were found to be motivated by the increase in the ambient stress in the shrimp. For example, Li et al. (2011) assessed the *GDH* gene expression alongside GDHase activity in the muscle of *L. vannamei* when fed dietary proteins. They found that both the GDHase activity and the *GDH* gene expression increased with higher dietary protein levels. When the shrimp were maintained at a different salinity, high GDHase activity was also recorded in *L. vannamei* after being fed a low-protein diet (Rosas et al., 2001). Contrary to the above results decreased GDHase activities were found in the muscles of almost all groups from both strains in this study. In addition, we found that the hepatic GDHase activity is much higher than the muscular enzyme activity and more quickly increase in hepatopancrease. We deduce that both increased GDHase activities and *GDH*-β gene expression could favor the reaction direction from glutamine and ${\text{NH}}_{4}^{+}$ to glutamate when the ammonia concentration increases (Figure 1; Plaitakis and Zaganas, 2001). Moreover, activated GDHase in the hepatopancreas plays an important role in metabolizing the excess ammonia.

After ammonia is decomposed by GDHase, the generated glutamate and excess ammonia are then assimilated into nitrogenous organic compounds by GSase catalytic functions in shrimp (Silvia et al., 2004; Teixeira and Fidalgo, 2009). This indicates that both GDHase and GSase play important roles in ammonia resistance and adaptation in shrimp. In *L. vannamei*, the GSase can participate in physiological osmotic adaptation to help resist acute salinity challenges. In most cases, both the GSase activities and the *GS* gene expression are induced in the hepatopancreas and the muscle in *L. vannamei* when exposed to stress (Liu et al., 2012). However, in this study, we found highly expressed *GDH* and *GS* genes only in the tissues of the two strains. According to the central dogma, we conclude that *L. vannamei* might first induce metabolic gene expression to address ammonia stress. In addition, contrary to muscle, the hepatic GSase activity was inhibited when the ambient ammonia stress increased in almost all groups. In terms of the relationship between enzymes and ammonia, we deduced that GDHase might play an important role in the hepatopancreas, while GSase is the key enzyme to regulate the ammonia balance in the muscle.

Studies have shown that both TGase and the *TG* gene play important roles in the immune deficiencies in *L. vannamei* (Huang et al., 2004; Wang et al., 2006). In a *TG* gene-silencing study, Fagutao et al. (2012) found that the absence of the *TG* gene might inhibit the regulation of the immune system in shrimp. However, the reactions of the TGase activity and the *TG* gene expression showed irregular trends in most cases when the ambient stress increased. For example, Guo et al. (2016) found that the *TG* gene was activated for 12 h, and then depressed from hour 12 to 72 when exposed to nitrite stress in the haemolymph of *L. vannamei*. The present study also indicated that high levels of ammonia might inhibit *TG* gene expression, while the TGase activity tended to be induced and then decreased in the haemocytes of *L. vannamei* (Chang et al., 2015). The TGase activities were mainly inhibited in strain B in both tissues when the ambient concentration of ammonia increased. Moreover, the *TG* gene expression mainly decreased in both tissues of strain A. These data indicated that a high level of ammonia stress might reduce ammonia metabolism rates by inhibiting the TGase activity firstly in the ammonia-resistant strain of *L. Vannamei*.

### Comparative analysis of selected biomarkers under ammonia stress between the two strains

While the IBR can be applied as a global index of environmental stress, the major challenge is to choose the related biomarkers to match a cause with an effect (Hagger et al., 2009; Oliveira et al., 2010). As a biomonitoring organism, *L. vannamei* is commonly used to assess the bioavailability and impact of contaminants in marine environments (Keating et al., 2007). Wang et al. (2012) suggested that the incorporation of biomarkers with the analysis of an IBR can be a useful tool for the identification of toxic contaminants in *L. vannamei*. Here, the total IBR index fit the strain tolerability accurately in both the hepatopancreas and the muscle with the increase in ammonia. This result indicated that the combination of these biomarkers could provide visual stress distinctions between GDHase, GSase, and TGase activities in *L. vannamei*. In addition, the data suggest that the results of the IBR analysis consider the relevance of the selected biomarkers rather than the selected abundance. These findings indicate that biomarkers associated with contaminants could clearly demonstrate the biological toxicity. The integrated biomarkers of GDHase, GSase, and TGase can provide more sensitive information and enhance the ability to detect the early signs of ammonia stress in shrimp.

## Conclusion

To respond ammonia stress, *L. vannamei* could induce activity of the catabolic enzyme (GSase) and inhibit the productive enzyme (TGase) activity to maintain ammonia balance in the muscle of the two strains. Compared with strain A, strain B could stimulate the activity of ammonia metabolizing enzymes in an earlier stage, which plays an important role in maintaining the stability of the content of ammonia-N in shrimp. The IBR demonstrated that, compared to the muscle, the hepatopancreas is more sensitive to ammonia stress and the hepatic tissue might be the main tissue that causes the different responses in the two strains. Biomarker responses were significantly correlated with ammonia concentrations and strain susceptibility in tissues, strongly suggesting a clear causal relationship and indicating that GDHase, GSase, and TGase can be used as biomarkers to monitor ammonia stress in *L. vannamei*. As for the gene expression, the data shows that the *L. vannamei* with high adaptability (strain B) could adapt to ammonia stress by expressing ammonia decomposing genes via *GDH*-β and *GS* as well as inhibiting hepatic ammonia synthesis gene expression via *TG*. This study provides useful data on *L. vannamei* under ammonia stress and might help reveal the physiological responses mechanism of ammonia-N in diverse strains.

## Author contributions

HZ, XD: Conceived and designed the experiments; LQ and SY: Carried out the experiments and analyzed the data; HZ: Supervised the project; LQ: Wrote and the manuscript; QH: Revised the manuscript, and all authors reviewed the manuscript.

### Conflict of interest statement

The authors declare that the research was conducted in the absence of any commercial or financial relationships that could be construed as a potential conflict of interest.
